# *Lactobacillus helveticus*-Derived Whey-Calcium Chelate Promotes Calcium Absorption and Bone Health of Rats Fed a Low-Calcium Diet

**DOI:** 10.3390/nu16081127

**Published:** 2024-04-11

**Authors:** Wei Hu, Zhiwen Pei, Aonan Xia, Yang Jiang, Bo Yang, Xiaoming Liu, Jianxin Zhao, Hao Zhang, Wei Chen

**Affiliations:** 1 State Key Laboratory of Food Science and Technology, Jiangnan University, Wuxi 214122, China; 6210112027@stu.jiangnan.edu.cn (W.H.); 6210113068@stu.jiangnan.edu.cn (Z.P.); 7210112107@stu.jiangnan.edu.cn (A.X.); 7160112009@vip.jiangnan.edu.cn (Y.J.); bo.yang@jiangnan.edu.cn (B.Y.); jxzhao@jiangnan.edu.cn (J.Z.); zhanghao@jiangnan.edu.cn (H.Z.); chenwei66@jiangnan.edu.cn (W.C.); 2 School of Food Science and Technology, Jiangnan University, Wuxi 214122, China; 3 National Engineering Research Center for Functional Food, Jiangnan University, Wuxi 214122, China

**Keywords:** whey-calcium chelate, low-calcium diet, calcium absorption, bone health

## Abstract

This study investigated the characteristics of *Lactobacillus helveticus*-derived whey-calcium chelate (LHWCC) and its effect on the calcium absorption and bone health of rats. Fourier-transform infrared spectroscopy showed that carboxyl oxygen atoms, amino nitrogen atoms, and phosphate ions were the major binding sites with calcium in LHWCC, which has a sustained release effect in simulated in vitro digestion. LHWCC had beneficial effects on serum biochemical parameters, bone biomechanics, and the morphological indexes of the bones of calcium-deficient rats when fed at a dose of 40 mg Ca/kg BW for 7 weeks. In contrast to the inorganic calcium supplement, LHWCC significantly upregulated the gene expression of transient receptor potential cation V5 (TRPV5), TRPV6, PepT1, calcium-binding protein-D9k (Calbindin-D9k), and a calcium pump (plasma membrane Ca-ATPase, PMCA1b), leading to promotion of the calcium absorption rate, whereas Ca_3_(PO_4_)_2_ only upregulated the TRPV6 channel in vivo. These findings illustrate the potential of LHWCC as an organic calcium supplement.

## 1. Introduction

Calcium mainly exists in the form of phosphates in bones and teeth, accounting for approximately 1.5% to 2% of the normal human body [1]. Calcium plays multiple physiological functions in the body and plays a crucial role in maintaining bone health. There is a greater demand for calcium in children and adolescents during their rapid growth period and the accumulation of calcium can achieve optimal peak bone mass in the early stages of life [2]. Although serum calcium can be maintained within the normal range through bone resorption, dietary intake of calcium is the only source of calcium supplementation in the body’s bones, and low calcium intake and bioavailability may lead to calcium deficiency. Long-term calcium deficiency in the body can lead to spasms, osteoporosis, and chondropathy. At present, calcium deficiency is a common global problem, and calcium intake in China is below the WHO’s daily recommended intake (1000 mg/d) [3], which calls for the attention of academia and the food industry in China.

In response to the current situation of calcium deficiency, calcium supplements such as inorganic calcium salts and organic calcium salts have been developed. Among them, organic calcium chelates such as amino acid-calcium chelates and protein hydrolysate-calcium chelates are known for their fast absorption rate and low energy consumption [4], with diversified sources such as soy protein hydrolysates [5], cucumber seed [6], casein hydrolysates [7], egg yolk hydrolysates [8], et al. The function of protein hydrolysate-calcium chelates has also been validated in vivo and in vitro [9]. Hua et al. [10] reported that after supplementation with *Chlorella pyrenoidosa* protein hydrolysate-calcium chelate (CPPH-Ca) to the male SD rats (3 weeks old), fed with the low-calcium diet, the physical and biomechanical properties of femurs and the gene expressions of transient receptor potential cation V5 (TRPV5), TRPV6, calcium-binding protein-D9k (Calbinding-D9k), and a calcium pump (plasma membrane Ca-ATPase, PMCA1b) in calcium-deficient rats were significantly improved by CPPH-Ca [10].

*Lactobacillus helveticus* is one of the commonly used lactic acid bacteria in the production of fermented dairy products such as yogurt and cheese [11]. It has a strong protein hydrolytic ability, which endows its fermented dairy products with rich and diverse bioactive peptide profiles with functions in the regulation of blood pressure [12], the immune system [13], cognition [14], et al. *L. helveticus* CCFM1263 has been shown to be highly efficient in casein hydrolysis and the generation of bioactive peptides [15]. The dairy protein hydrolysates of *L. helveticus* CCFM1263 would be a desirable agent for the development of functional calcium chelates. Therefore, in the present study, we aimed to investigate the characteristics of *Lactobacillus helveticus*-derived whey-calcium chelate (LHWCC) and its effect on the calcium absorption and bone health of rats fed with low-calcium diets.

## 2. Materials and Methods

### 2.1. Materials

Skim milk powder (lactose > 51.8%, protein > 35.8%) was provided by Bright Dairy Co., Ltd. (Shanghai, China). Chemicals, enzymes, and bile were purchased from Sigma Aldrich (Shanghai, China). Pepsin (Sigma P6887) and pancreatin (Sigma, P7545 8 USP) were of porcine origin, whereas bile (Sigma B8631) was of bovine origin. *Lactobacillus helveticus* CCFM1263 was isolated from naturally fermented dairy products in China and was deposited in the Culture Collection of Food Microorganisms (CCFM) of Jiangnan University (Wuxi, China). The AIN-93G rodent diet was purchased from Jiangsu Xietong Biology Co., Ltd. (Nanjing, Jiangsu, China). Nuclease-free water, FastPure cell/tissue total RNA isolation kit V2, HiScript III RT SuperMix for qPCR (+gDNA wiper), and ChamQ Universal SYBR qPCR Master Mix were purchased from Vazyme Biotech Co., Ltd. (Nanjing, Jiangsu, China). The primers for TRPV5, TRPV6, PepT1, Calbindin-D9k, Na^+^/Ca^2+^ exchange mechanism (NCX), PMCA1b and β-actin were compounded by the Genewiz Biotechnology Co., Ltd. (Suzhou, Jiangsu, China).

### 2.2. Preparation of Whey-Calcium Chelate

The preparation method described by Wang et al. [16] was used with moderate modifications. The strains were sub-cultured three times in MRS medium and then twice in sterile reconstituted skim milk (11% *w*/*w*) at 37 °C prior to use. After two washes in Tris-HCl (pH 6.5), 2% cultures with an initial concentration of 1–5 × 10^8^ CFU/mL were inoculated with sterile reconstituted skim milk (11% *w*/*w*) and incubated for 48 h at 37 °C. Then, 1 mol/L NaOH was added to adjust the sample to pH 4.6, heated at 95 °C for 10 min, and centrifuged. After centrifugation, the supernatant was filtered with 0.45 μM organic filter membrane and freeze-dried as lyophilized *Lactobacillus helveticus* whey (LHW). Then, LHW was dissolved in deionized water at a concentration of 3 mg/mL and mixed with CaCl_2_ at a Ca^2+^ concentration of 1 mg/mL. The solution was stirred at pH 7.6, 40 °C for 120 min [17]. Then, ethanol was added to remove free calcium from the samples. After centrifugation, the precipitate was freeze-dried to obtain LHWCC (125.85 ± 4.31 mg Ca/g).

### 2.3. Fourier-Transform Infrared Spectroscopy (FT-IR)

The FT-IR spectrum of LHW and LHWCC was recorded from 4000 to 400 cm^−1^ using a Nicolet Summit FT-IR spectrometer iS50 (Thermo Fisher Scientific Inc., Waltham, MA, USA).

### 2.4. In Vitro Digestion

In vitro digestion was based on the standardized COST INFOGEST protocol [18]. In the digestion test, samples were taken at the beginning and end of the oral phase, at 0, 15, and 120 min of simulated gastric juice digestion, and at 0, 15, 30, 60, and 120 min of simulated intestinal juice digestion. Then, the samples were centrifuged at 8000 r/min for 10 min at 4 °C, and the calcium concentration of the supernatant was measured.

### 2.5. Animal Experiments

**Figure 1:**
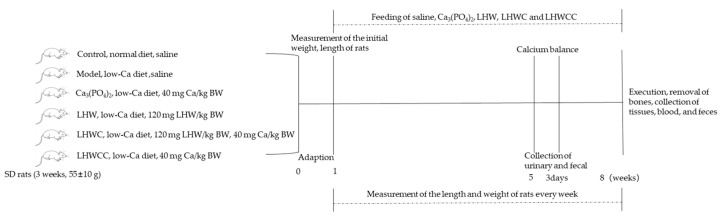
The flowchart of animal experiment. Note: the rats in the control group were fed the normal diet (5000 mg Ca/kg), and the remaining rats were fed a low-Ca diet (1000 mg Ca/kg).

Male SD rats (*n* = 48, 3 weeks old, 55 ± 10 g) were purchased from the Vital River Laboratory Animal Technology Co., Ltd. (Beijing, China). The animal experimental protocol was approved by the Animal Ethics and Welfare Committee of Jiangnan University (Wuxi, China), and the IACUC Issue No. was JN.No 20230530S1200730[247]. Animal feeding conditions met SPF level requirements, and during the entire experimental period, rats were free to eat commercial food, which was prepared according to the AIN-93 [19] (normal diet: 5000 mg Ca/kg; low-calcium diet: 1000 mg Ca/kg) [10], and drink freely. At the beginning of the experiment, the initial body length, body weight, and tail length were measured. During a 7-week experiment, the length and weight of rats were measured every week. The rats in the control group were fed the normal diet and the remaining rats were fed a low-Ca diet. The rats were randomly assigned into six groups, and the details of the experimental design are shown in Figure 1.

### 2.6. Analysis of Serum Biochemical Indexes

The serum levels of calcium and ALP in different groups of rats were analyzed with analytical reagent kits (Nanjing Jiancheng Bioengineering Institute, Nanjing, China).

### 2.7. Analysis of Femur Length, Diameter, and Weight

The femurs that were cleaned of soft tissue were placed in a constant weight box and dried thoroughly in the drying oven for 10 h until reaching constant weight (under the condition that the lid is opened at 80 °C for 6 h and then closed at 115 °C for 4 h). Then, the dry weight of the femur was weighed and the length of the femur was measured. The diameter from the descending third rotor to the junction of the femoral shaft was determined as the femoral diameter.

### 2.8. Analysis of Calcium Content in Femur

After being dried, a 100 mg femur was dissolved in 7 mL of 16 mol/L HNO_3_ to determine calcium content by flame atomic absorption spectrometry (AA-240, Varian Medical Systems, Palo Alto, CA, USA). The atomic absorbance was monitored at 422.7 nm, and the Ca content in the femur was expressed on a mg/g dry basis [20].

### 2.9. BMD and Bone Mass Measurements

The BMD of the femur was measured using a micro-CT (PerkinElmer Quantum GX, Waltham, MA, USA). Bone and trabecular-related indexes of the proximal femoral were obtained using the built-in software of the micro-CT. The operation parameters were as follows: 90 kV tube potential, 88 μA tube current, 86 mm FOV, high-resolution scan mode, 4 min scanning time, and 0.1 mm Cu X-ray filter according to the method of Chen et al. [21]. Calculations included bone volume (BV), bone surface (BS), bone volume/tissue volume (BV/TV), bone surface/tissue volume (BS/TV), trabecular separation (Tb.Sp), trabecular thickness (Tb·Th), and connectivity density (Conn.D).

### 2.10. Bone Biomechanical Strength Measurements

A three-point bending mechanical test was performed on the left femur diaphysis using a TA-XT plus texture analyzer (Stable Micro Systems, Godalming, Surrey, UK). The maximum fracture force, i.e., the maximum bone load, was measured using a three-point bending test at a test speed of 1 mm/s. The test was performed with a fulcrum span of 16 mm according to the method of Ye et al. [22] with slight modification.

### 2.11. Analysis of Calcium-Apparent Absorption and Retention Rate

The calcium intake and fecal and urinary calcium content were determined by AAS during the last 3 days of treatment. The calcium metabolism was calculated according to Wang et al. [23] with the following formula:

Apparent calcium absorption rate (ACAR) (%) = (Calcium Intake − Fecal Calcium)/Calcium Intake × 100%.(1)

Calcium accumulation rate (CAR) (%) = (Calcium Intake − Fecal Calcium − Urinary Calcium)/Calcium Intake × 100%.(2)

### 2.12. RNA Extraction and Real-Time RT-PCR

The intestinal tissue was ground and the RNA was obtained according to the instructions of the total RNA extraction kit. The nucleic acid purity (OD260/280 = 1.8–2.2, OD260/230 ≥ 2.0) and concentration were tested. The samples were reverse transcribed into cDNA according to the instructions of the reverse transcription kit. The primer reference was Li et al. [6], and the gene primer sequence was shown in Table 1. β-Actin was selected as the housekeeping gene. Relative gene expressions were calculated by the comparative 2^−ΔΔCt^ method.

### 2.13. Statistical Analysis

Data for each group were expressed as mean ± SD (*n* = 8). For data analysis, one-way analysis of variance (ANOVA) was conducted using IBM SPSS Statistics 26 software (SPSS Inc, Chicago, IL, USA). Graphing and data processing were performed using GraphPad Prism 9.0.

## 3. Results

### 3.1. Fourier Transform-Infrared Spectroscopy of LHWCC

**Figure 2:**
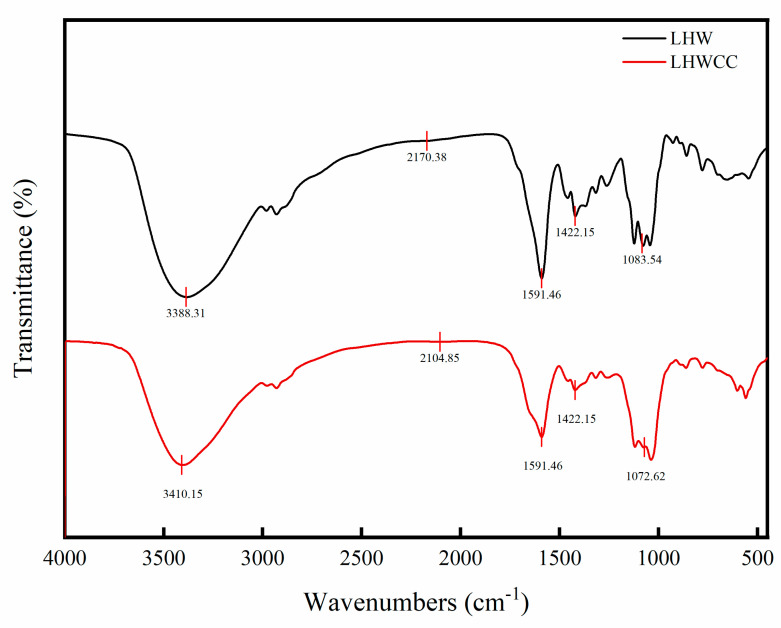
Fourier transform-infrared spectra of LHW and LHWCC in the regions of 4000 to 400 cm^−1^.

The infrared spectra of LHW and LHWCC in the wavelength range of 400–4000 cm^−1^ are shown in Figure 2, and the infrared spectrum of the calcium chelate showed significant changes. LHWCC had obvious fluctuations at 3410 cm^−1^, 2104 cm^−1^, 1591 cm^−1^, 1422 cm^−1^, and 1072 cm^−1^. The characteristic peaks of amide A and amide B bands in the sample at 3388 cm^−1^ are significantly weakened and shifted, which was caused by the inductive effect or the dipole field effect, indicating the binding of calcium ions with N-H [24]. The absorption peak of LHW at 2170 cm^−1^ corresponding to the phosphate group O=P-O-H disappeared after chelation, indicating that H in the phosphate group was replaced by calcium ions. The absorption peak of LHWCC weakened at 1600 cm^−1^ and 1400 cm^−1^, indicating that -COOH participated in the formation of chelates in the form of covalent bonds [25] with calcium ions to form -COO-Ca. The absorption peak of LHWCC shifted at 1072 cm^−1^, which might be due to the formation of C-O-Ca by the combination of -CO bond and calcium [26]. The changes in absorption peaks in the range of 800–500 cm^−1^ could be attributed to the effect of chelation on the vibration of C-H and N-H bonds in carboxyl oxygen atoms, amino nitrogen atoms, and phosphate ions in compounds such as peptides in LHWCC.

### 3.2. Soluble Calcium Content of LHWCC In Vitro Digestion

**Figure 3:**
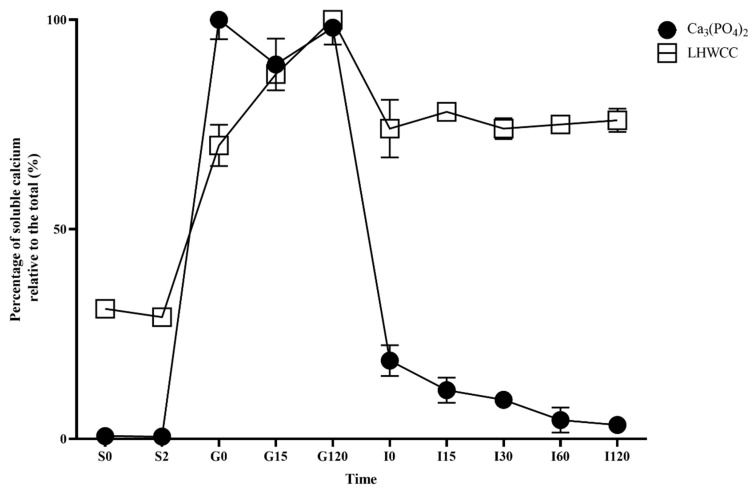
Changes in percentage of soluble calcium (relative to the total of calcium) for Ca_3_(PO_4_)_2_ and LHWCC during in vitro digestion.

As shown in Figure 3, there were significant differences in the dissolution characteristics of different calcium agents during the simulated oral digestion stage. Ca_3_(PO_4_)_2_ was almost insoluble in the simulated saliva, and the solubility of LHWCC was around 30%. In the simulated gastric digestion stage, the solubility of both calcium agents significantly increased as an acidic environment (pH 3.0) increased calcium solubility [9]. Ca_3_(PO_4_)_2_ was almost completely dissolved when entering the simulated gastric juice and subsequently maintained a solubility of over 90%. The solubility of LHWCC during gastric digestion increased with time, from the initial 70% to 100%, which was consistent with grape seed polypeptide calcium chelate [27]. This indicated that LHWCC has a certain sustained-release effect, which can reduce the rapid release of calcium ions in the stomach.

After entering the intestinal buffer, the solubility of Ca_3_(PO_4_)_2_ rapidly decreased to 20% and gradually decreased to 3% with time. On the other hand, the solubility of LHWCC slightly decreased to 70% when entering the alkaline environment of the intestine and remained stable afterward.

### 3.3. Effect of LHWCC on Weight, Body Length, and Tail Length Gain

Figure S1 shows the weight changes in rats during the experiment. The weight of rats in different treatment groups showed steady growth, and there was no significant difference among the groups in body weight and length (*p* > 0.05). The low calcium treatment resulted in slow tail growth. The treatments with LHW, co-administration of Ca_3_(PO_4_)_2_ with LHW (LHWC), and LHWCC significantly increased the tail length of rats, whereas Ca_3_(PO_4_)_2_ had no significant effect on tail length compared to the model group.

### 3.4. Effect of LHWCC on Femoral and Serum Indicators

**Figure 4:**
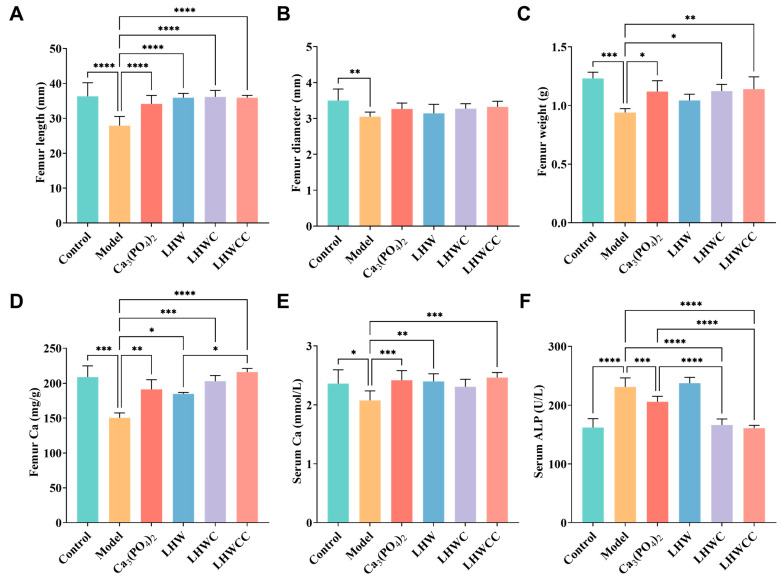
Changes in the bone morphology and calcium content and serum biochemical parameters of rats (**A**), femur length (**B**), femur diameter (**C**), femur weight (**D**), femur calcium (**E**), serum calcium (**F**), serum ALP. Data are expressed as the mean ± SD (*n* = 8). * *p* < 0.05; ** *p* < 0.01; *** *p* < 0.005; **** *p* < 0.001.

Figure 4A–F summarizes the changes in femoral and serum biochemical parameters. A low-calcium diet led to a significant decrease in bone length (*p* < 0.001), bone diameter (*p* < 0.01), bone weight (*p* < 0.005), and bone and serum calcium content (*p* < 0.005), with a significant increase in ALP activity (*p* < 0.005). Treatments with Ca_3_(PO_4_)_2_ and LHWCC significantly increased bone length, as well as bone and serum calcium content, with no significant difference in most indicators among the groups fed with Ca_3_(PO_4_)_2_, LHWC, and LHWCC (*p* > 0.05). Ca_3_(PO_4_)_2_, LHWC, and LHWCC treatment resulted in significantly lower ALP activity compared to the model group (*p* < 0.001), with a stronger effect of LHWC and LHWCC on ALP activity than that of the Ca_3_(PO_4_)_2_ (*p* < 0.001). LHW had no effect on serum ALP activity in rats and the weight of the femur (*p* < 0.05), whereas the intake of LHW had an impact on both femur and serum calcium content. These results indicated that supplementation of LHW was beneficial for restoring femoral morphology and calcification in the state of low calcium, whereas LHWCC was as effective in the improvement of the serum and femoral indicators as Ca_3_(PO_4_)_2,_ and even more effective in the improvement of ALP activity in calcium-deficient rats.

### 3.5. Bone Biomechanical Parameters and Histomorphometry

**Figure 5:**
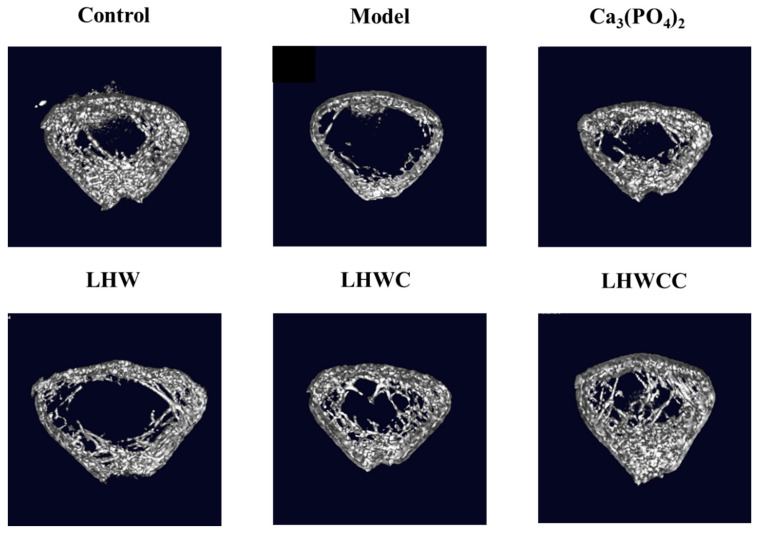
Three-dimensional micro-CT images of the distal femur of rats.

The three-dimensional (3D) reconstruction of micro-CT images of the left femoral trabecular bone is shown in Figure 5. Compared with the control group, the bone trabeculae of low-calcium rats were sparse, with large gaps and obvious damage in their morphology and network connectivity structure. Supplementation with Ca_3_(PO_4_)_2_, LHWC, and LHWCC all resulted in a significant effect on the bone trabeculae and an increase in thickness and connectivity, with LHWCC as the improvement of the microstructure of bone trabeculae and enhancing bone quality. On the other hand, LHW only showed a weak influence on the morphology of bone trabeculae.

**Figure 6:**
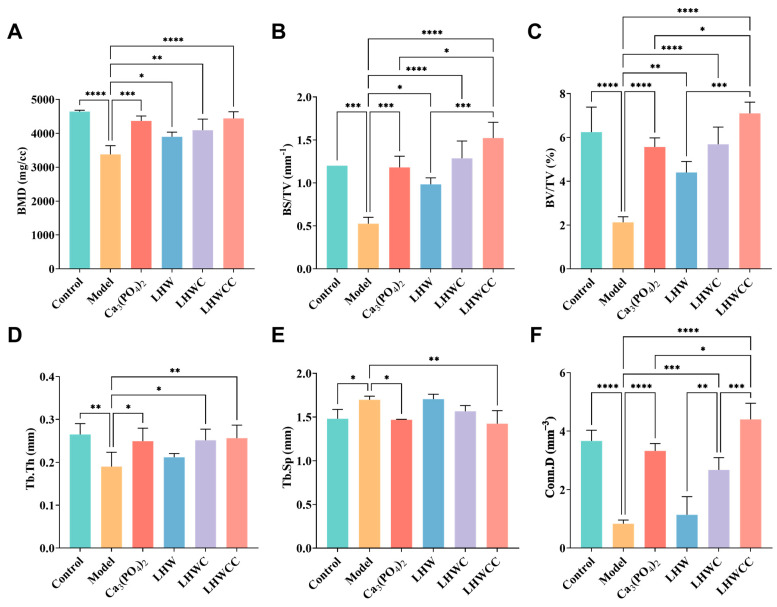
Changes in BMD and bone mass of rats (**A**), BMD (**B**), BS/TV (**C**), BV/TV (**D**), Tb.Th. (E), Tb.Sp (**F**), Conn.D. Data are expressed as the mean ± SD (*n* = 8). * *p* < 0.05; ** *p* < 0.01; *** *p* < 0.005; **** *p* < 0.001.

The results of bone mass are shown in Figure 6. Low calcium treatment resulted in a significant decrease in femoral bone density (*p* < 0.001), BS/TV (*p* < 0.001), and BV/TV (*p* < 0.005) in rats (Figure 6A–C). After treatment, femoral BMD improved significantly in all groups, and the BMD of rats in LHWCC group was the highest. Bone mass was remarkably recovered with the intervention of Ca_3_(PO_4_)_2_, LHW and LHWC, and LHWCC, with LHWCC showing a stronger regulating effect on bone loss than Ca_3_(PO_4_)_2_ (*p* < 0.05).

Low calcium treatment significantly reduced the thickness and the connectivity density (*p* < 0.001) and increased the separation of bone trabeculae (*p* < 0.05) (Figure 6D–F) as indicated by indexes such as Th.Tb (an indicator of the thickness of the trabecular bone), Th.Sp (an indicator of the bone trabecular separation), and Conn.D (an indicator of the degree of trabecular bone separation). Both Ca_3_(PO_4_)_2_ and LHWCC showed significant effects on promoting bone trabeculae. The coadministration of LHW and Ca_3_(PO_4_)_2_ had a significant effect on restoring Th.Tb (*p* < 0.05) and Conn.D (*p* < 0.005), with no effect on Th.Sp, whereas no influence on bone trabeculae was recorded by LHW treatment.

### 3.6. Effects on Bone Biomechanical Strength

**Figure 7:**
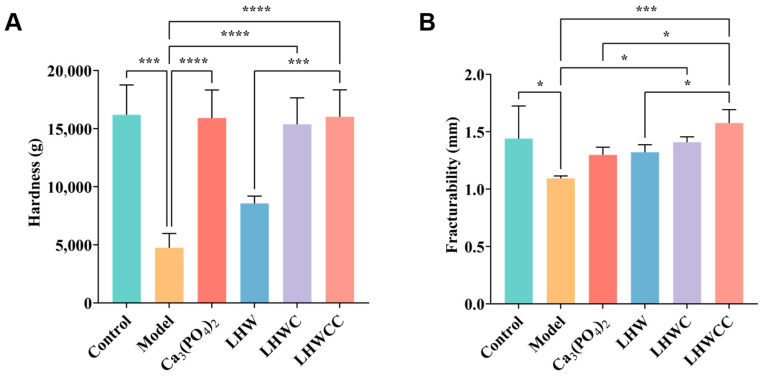
Changes in bone biomechanical property of rats (**A**), hardness (**B**), fracturability. Data are expressed as the mean ± SD (*n* = 8). * *p* < 0.05; *** *p* < 0.005; **** *p* < 0.001.

From Figure 7, it can be seen that low calcium intake resulted in significant decreases in the maximum bone load (*p* < 0.005) and maximum deflection. Supplementation with Ca_3_(PO_4_)_2_, LHWC, and LHWCC significantly improved bone biomechanics (*p* < 0.001) in rats, whereas LHW showed no effect. There was no significant difference between the regulating effect of Ca_3_(PO_4_)_2_ and LHWCC on hardness, whereas LHWCC performed better than Ca_3_(PO_4_)_2_ in fracturability.

### 3.7. Effect of LHWCC on Calcium Balance

**Figure 8:**
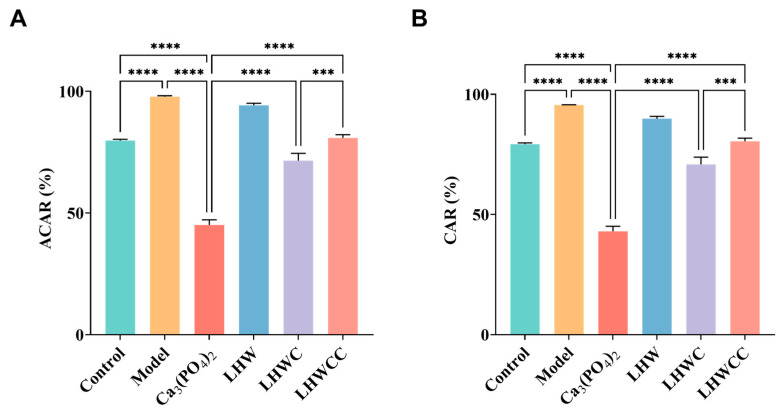
Changes in calcium metabolism of rats (**A**), apparent calcium absorption rate (ACAR) (**B**), calcium accumulation rate (CAR). Data are expressed as the mean ± SD (*n* = 8). *** *p* < 0.005; **** *p* < 0.001.

From Figure 8, it can be seen that the apparent calcium absorption rate (ACAR) and calcium accumulation rate (CAR) of the model group were significantly increased compared to the control group (*p* < 0.001), which might be due to pathological compensation formed to maintain calcium concentration in the body in a calcium-deficient state [5]. Ca_3_(PO_4_)_2_ treatment significantly decreased both ACAR and CAR. Although LHW treatment did not result in changes in calcium balance, the combination of Ca_3_(PO_4_)_2_ with LHW showed higher calcium absorption rates than that of Ca_3_(PO_4_)_2_ alone (*p* < 0.001), indicating that LHW could promote the absorption and accumulation of inorganic calcium in rats. When LHW was chelated with calcium, its apparent absorption rate increased to 80%, indicating that the chelation had a beneficial effect on calcium absorption and retention in calcium-deficient rats compared to inorganic calcium in the body [28], which is consistent with the pattern of calcium release of Ca_3_(PO_4_)_2_ and LHWCC during in vitro digestion.

### 3.8. Gene Expression of Corresponding Receptors in the Intestines of Rats

**Figure 9:**
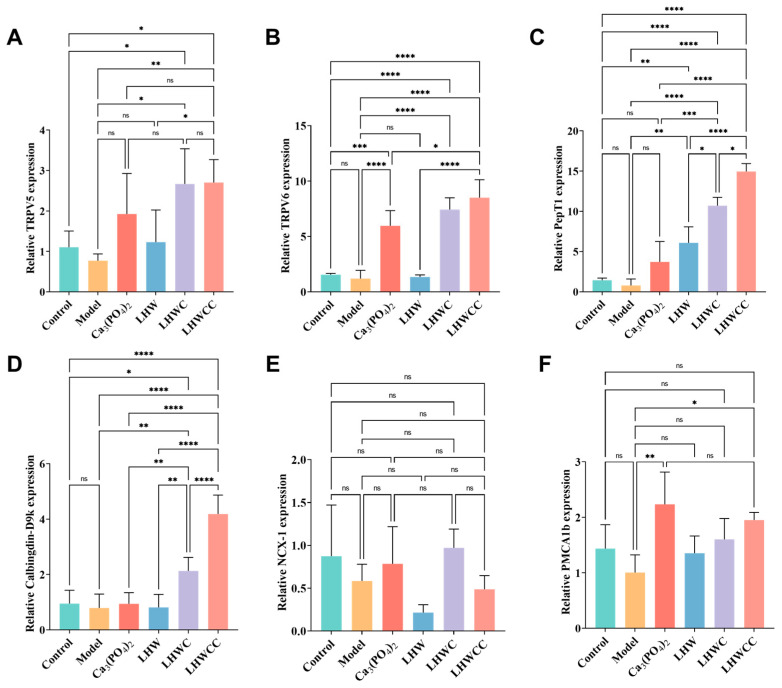
Calcium absorption-related gene expression in intestines of rats (**A**), TRPV5 (**B**), TRPV6 (**C**), PepT1 (**D**), Calbinding-D9k. (**E**), NCX-1 (**F**), PMCA1b. Data are expressed as the mean ± SD (*n* = 8). ns *p* > 0.05; * *p* < 0.05; ** *p* < 0.01; *** *p* < 0.005; **** *p* < 0.001.

The expression of calcium absorption-related genes in the small intestine of rats is shown in Figure 9, and the inorganic calcium salt and organic chelates varied in the regulation of the related gene expression. The expression levels of TRPV5, TRPV6, PepT1, and Calbindin-D9k significantly increased by LHWC and LHWCC treatments, whereas Ca_3_(PO_4_)_2_ showed notable elevation of the expression of TRPV6 and PMCA1b. In addition, compared to the control, LHWCC showed increased expression levels of TRPV5/6, PepT1, and Calbindin D9k. Among all the treatments, LHWCC showed the strongest regulating effect. On the other hand, LHW only significantly increased the expression of PepT1. However, none of the treatments had an impact on the expression of NCX-1.

## 4. Discussion

The FT-IR spectra were used to provide more information on the binding of metal ions with organic ligand groups of LHW, showing that the main binding sites of LHWCC included carboxyl oxygen atoms, amino nitrogen atoms, and phosphate ions, which was similar to CPPH-Ca [10].

Calcium in food can be dissolved into ionic form through gastric acid digestion but it is prone to precipitate in the relatively alkaline environments of the small intestine, leading to a decrease in bioavailability [29]. In vitro models must be created to investigate the calcium release patterns of different calcium supplements [30]. Compared to Ca_3_(PO_4_)_2_, LHWCC was less affected by pH, which was similar to the in vitro digestion results of CPP-Ca [31]. Jiang et al. [27] found that most peptide calcium chelates were soluble after digestion, but the dialysis rate was significantly lower than the solubility. Therefore, it is speculated that calcium ions remained in a binding state to macromolecules during in vitro digestion. The pattern of sustained release of calcium and high solubility of LHWCC during simulated intestinal digestion might be partially attributed to the presence of peptides in LHW [15,32] as supported by the upregulation of PepT1 in vivo. Anyway, previous research found that during the simulated in vitro digestion process of the intestine, the solubility of CaCO_3_ was between 60 and 80% [9], whereas that of Ca_3_(PO_4_)_2_ was below 20%, with significant differences in solubility. The low solubility constant of Ca_3_(PO_4_)_2_ led to its low calcium solubility during simulated intestinal digestion. However, in vivo experiments showed that the apparent absorption rate in rats of CaCO_3_ was about 40% [33], which is close to Ca_3_(PO_4_)_2_ (45%) in the present study. Our results also indicated that among the calcium absorption-related genes analyzed in the present study, Ca_3_(PO_4_)_2_ only upregulated the expression of the TRPV6 channel, similar to the previous study on CaCO_3_ [34,35]. Therefore, although the two inorganic calcium salts Ca_3_(PO_4_)_2_ and CaCO_3_ had significant differences in solubility in vitro, they shared similarities in both the effect and mechanism of the regulation of calcium absorption in vivo.

Low-calcium diets may cause microarchitectural deterioration of bone tissue, leading to increased bone fragility and risk of fracture [36]. The present study found that Ca_3_(PO_4_)_2_, LHWC, and LHWCC significantly restored serum biochemical parameters, bone morphology, and the bone biomechanical property of low-calcium-fed rats, whereas LHW only upregulated the PepT1 expression level to improve serum and bone calcium content in rats. Chelates such as LHWCC showed a stronger influence on trabecular microstructure and calcium absorption than Ca_3_(PO_4_)_2_ and the co-administration of LHW and Ca_3_(PO_4_)_2_. Bone microstructure, especially trabecular microstructure, plays an important role in monitoring calcium deposition in bone and characterizing bone growth and development levels [37,38]. Bone trabeculae have a certain shape and distance in the bone marrow cavity, cross-linking with each other to form a network structure, and are mainly responsible for maintaining bone strength, bearing loads, and hematopoietic functions [39]. Compared with Ca_3_(PO_4_)_2_ and LHWC, LHWCC increased bone mass by promoting the number and thickness of bone trabeculae and making the cross-linking of bone trabeculae denser, highlighting the improvement of efficiency of promotion of bone health through the conversion of inorganic calcium supplements to organic agents, as previously reported [33,40,41].

**Figure 10:**
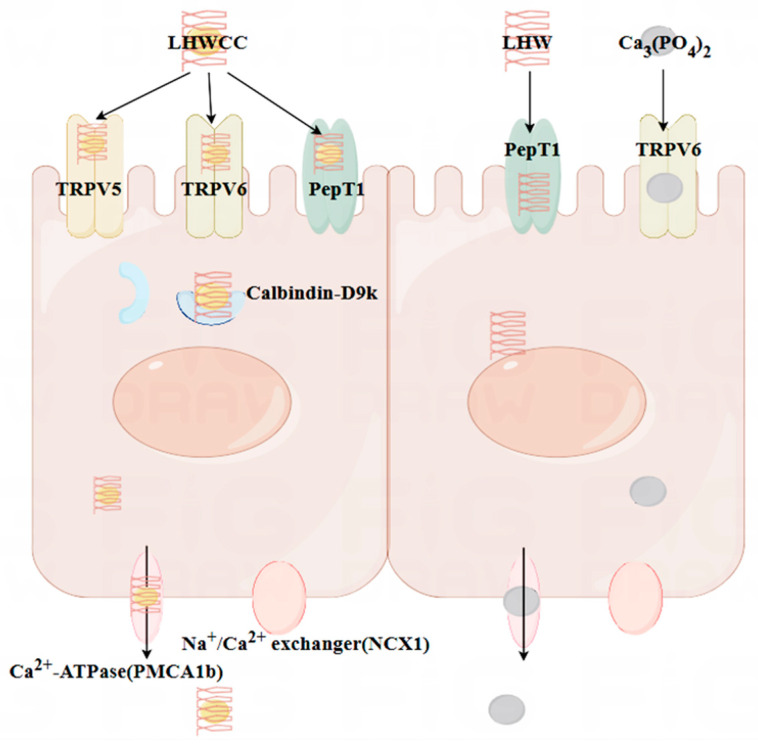
The mechanistic interpretation of intestinal calcium absorption of different calcium agents investigated in the present study.

The small intestine is the main organ responsible for calcium absorption, responsible for over 90% of calcium absorption in the human body [29]. The process of calcium absorption in intestinal cells can be roughly divided into three steps: calcium ions enter the cells through TRPV5/6; calcium binds to Calbindin-D9k for intracellular transport; it is pumped into the bloodstream by NCX-1 and PMCA1b. The main rate-limiting step of the above process is to absorb calcium into cells, which means that the efficiency of active calcium transport is mainly related to channel proteins such as TRPV5/6 [7]. As shown in Figure 10, both LHWCC and CPPH-Ca exerted promotive effects on calcium absorption via regulation of the gene expression of TRPV5, TRPV6, Calbinding-D9k, and PMCA1b. In addition, it is reported that peptide calcium chelates can resist gastric digestion as short peptides enter the intestine. The main pathways currently known to transport peptides through the intestinal cells are the PepT1 pathway and the cell-penetrating peptide pathway, both of which are transcellular pathways. The PepT1 pathway is a widely specific peptide transporter protein that can transport almost all dipeptides and tripeptides [4]. The results have shown that LHWCC also regulated the higher expression level of PepT1 than Ca_3_(PO_4_)_2_ and LHWC, which would facilitate the transition of peptides into the intestine and contribute to the enhancement of calcium absorption indirectly due to the increased proportion of oligopeptides in protein hydrolysates after chelating with calcium [42]. Therefore, among the various calcium chelates, protein hydrolysate-calcium chelates are unique as they could upregulate not only the pathway of calcium absorption such as TRPV5, TRPV6, and Calbinding-D9k, but also an indirect pathway potentially beneficial for bone health via PepT1.

On the other hand, LHWCC significantly increased calcium absorption in vivo compared to Ca_3_(PO_4_)_2_, which may be due to LHWCC significantly upregulating the gene expression of TRPV5 and TRPV6, whereas calcium phosphate can only significantly upregulate the TRPV6 pathway. The expression level of Calbindin-D9k was also higher in LHWCC. TRPV5 and TRPV6 have 75% homology and their main differences are in the N and C terminal tails. Both channels permeate calcium ions, and TRPV5 exhibits stronger ion selectivity. TPPV6 can be regulated by vitamin D_3_, as well as Calbindin-D9k and PMCA1b, which may be regulated through interactions with these calcium transporters and enzymes involved in intestinal calcium absorption and increased parathyroid hormone (PTH) [43,44]. Meanwhile, PTH also indirectly affects the content of 1,25(OH)_2_D_3_, the hormonal form of vitamin D [45]. Calbindin-D9k is regulated at the transcriptional and post-transcriptional levels by the serum level of 1,25(OH)_2_D_3_ [46]. Therefore, the increased expression levels of TRPV6 and Calbindin-D9k in LHWCC may be related to PTH and 1,25(OH)_2_D_3_ in the serum of rats. Further experiments in this aspect would be desirable for understanding the mechanism of the effect of LHWCC on calcium absorption and bone health.

## 5. Conclusions

In conclusion, LHWCC exerted a notable impact on bone health. Compared with inorganic calcium, LHWCC had a higher solubility during in vitro digestion and had promotive effects on serum biochemical parameters, bone microstructure, and calcium absorption in vivo. LHWCC treatment exerted promotive effects on calcium absorption by upregulating TRPV6, TRPV5, PepT1, Calbindin-D9k, and PMCA1b-signaling pathways in intestines, thereby improving serum and bone calcium concentration and restoring bone biomechanical parameters and histomorphometry.

## Figures and Tables

**Table 1:** Primers used in the measurement of mRNA expression.

Gene	Primer Sequence
TRPV6	forward 5′-CACCCAGTGGACGTATGGAC-3′
	reverse 5′-CTCGTGCGGTTATTGGTCCT-3′
TRPV5	forward 5′-ACGTATGGACCCCTGACCTC-3′
	reverse 5′-GAATTTGGCGAGCCTCTCGT-3′
Calbindin-D9K	forward 5′-GGCAACCAGACACCAGAATGA-3′
	reverse 5′-TGACAACTGGTCTGGATCACC-3′
NCX-1	forward 5′-TTGAGATTGGAGAACCCCGT-3′
	reverse 5′-ATGTGAAGCCACCAAGCTCA-3′
PMCA1b	forward 5′-AGTGATTGTTGCTTTTACGGGC-3′
	reverse 5′-AGAGACTCAGTGGGTGGTTCCG-3′
PepT1	forward 5′-ATCTACCATACGTTTGTTGC-3′
	reverse 5′-CTGGGGCTGAAACTTCTT-3′
β-Actin	forward 5′-CACCCAGCACAATGAAGATCAAGAT-3′
	reverse 5′-CCAGTTTTTAAATCCTGAGTCAAGC-3′

## Data Availability

Data are contained within the article and Supplementary Materials.

## Supplementary Materials

The following supporting information can be downloaded at: https://www.mdpi.com/article/10.3390/nu16081127/s1, Figure S1: changes in body weight, body length, and tail length of rats (A), weight (B), body length (C), tail length.
