# Supplementary material for: Lactobacillus helveticus-Derived Whey-Calcium Chelate Promotes Calcium Absorption and Bone Health of Rats Fed a Low-Calcium Diet
Source: Nutrients. 2024 Apr 11;16(8):1127. doi: 10.3390/nu16081127 (PMC11053418; doi:10.3390/nu16081127)
Supplement: Supplementary file 1 [file nutrients-16-01127-s001.zip › nutrients-2946962-supplementary.pdf]

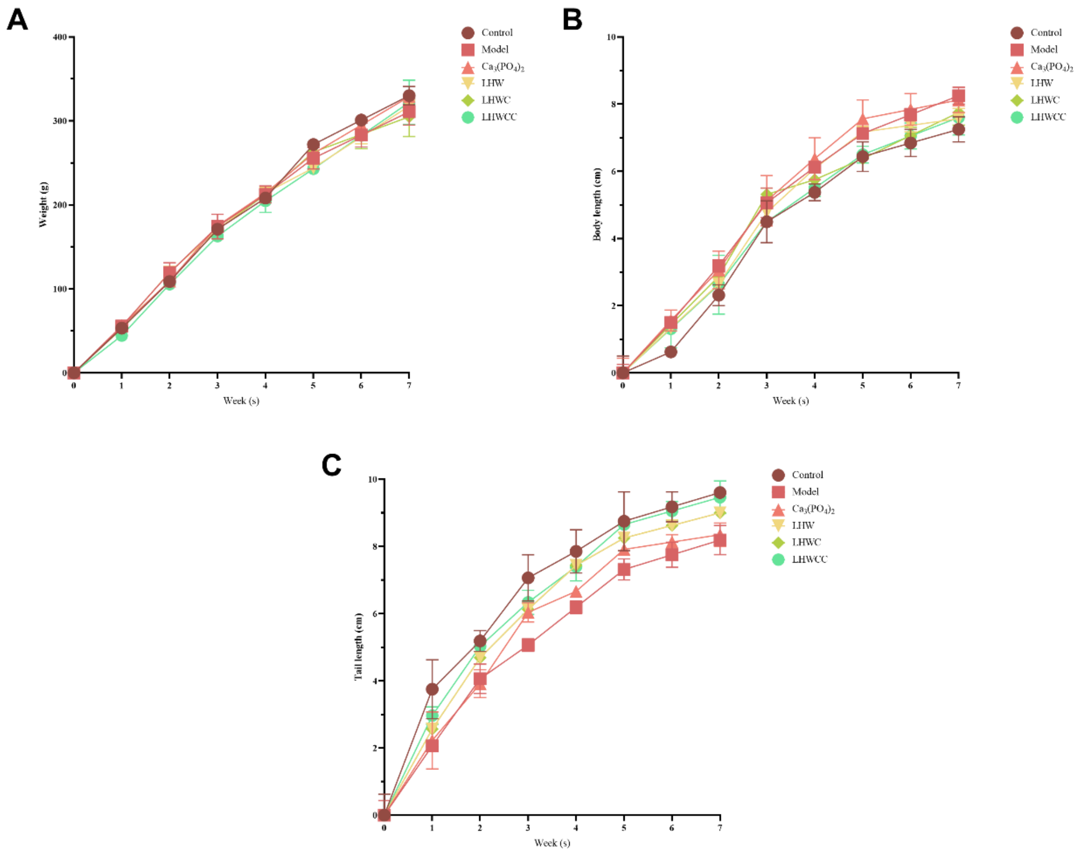

Figure S1 Changes in body weight, body length and tail length of rats (A), weight (B), body length (C), tail length.
